# The international nucleotide sequence database collaboration

**DOI:** 10.1093/nar/gkaa967

**Published:** 2020-11-09

**Authors:** Masanori Arita, Ilene Karsch-Mizrachi, Guy Cochrane

**Affiliations:** Bioinformation and DDBJ Center, National Institute of Genetics, Mishima, Shizuoka 411-8540, Japan; National Center for Biotechnology Information, National Library of Medicine, National Institutes of Health, Bethesda, MD 20894, USA; European Molecular Biology Laboratory, European Bioinformatics Institute (EMBL-EBI), Wellcome Genome Campus, Hinxton, Cambridge CB10 1SD, UK

## Abstract

The International Nucleotide Sequence Database Collaboration (INSDC; http://www.insdc.org/) has been the core infrastructure for collecting and providing nucleotide sequence data and metadata for >30 years. Three partner organizations, the DNA Data Bank of Japan (DDBJ) at the National Institute of Genetics in Mishima, Japan; the European Nucleotide Archive (ENA) at the European Molecular Biology Laboratory's European Bioinformatics Institute (EMBL-EBI) in Hinxton, UK; and GenBank at National Center for Biotechnology Information (NCBI), National Library of Medicine, National Institutes of Health in Bethesda, Maryland, USA have been collaboratively maintaining the INSDC for the benefit of not only science but all types of community worldwide.

## INTRODUCTION

The International Nucleotide Sequence Database Collaboration (1) (INSDC; http://www.insdc.org/) is the core infrastructure for sharing nucleotide sequence data (NSD) and their subsidiary information called metadata in the public domain. The collaboration is comprised of three nodes that keep the identical information through a daily data exchange process that has operated for over 30 years:

- the DNA Data Bank of Japan (DDBJ; http://www.ddbj.nig.ac.jp/) at the National Institute of Genetics in Mishima, Japan (2);
- the European Nucleotide Archive (ENA; http://www.ebi.ac.uk/ena/) at the European Molecular Biology Laboratory's European Bioinformatics Institute (EMBL-EBI) in Hinxton, UK (3); and
- GenBank (https://www.ncbi.nlm.nih.gov/genbank/) at National Center for Biotechnology Information (NCBI), National Library of Medicine, National Institutes of Health in Bethesda, Maryland, USA (4).

The scope of INSDC is not limited to NSD (Table 1). Information about research projects and physical biomaterials are collected as BioProject and BioSample records (4), respectively, with links to NSD. Raw data and alignment information from next-generation sequencers are stored separately from the assembled sequence data in the traditional, annotated archives; naming of, and links to, these INSDC components at each partner site are provided in Table 1 and at http://www.insdc.org/. The key links across these databases are Accession Numbers (ANs), i.e., the unique and permanent identifiers issued by the INSDC for each submitted sequence. In the vast majority of life science and medical journals, reporting of ANs is mandatory for sequence studies, and relationships with journal publishers have been established to guarantee the data accessibility and to assist reproducibility of published results. The INSDC functions as the unique registry of all publicly available NSD.

**Table 1.** Databases of the three INSDC nodes and starting years. The four left columns indicate INSDC resources and the two right columns are associated repositories which are not formally part of the INSDC exchange

	Annotated sequences	NGS reads	Project metadata	Sample information	Functional genomics	Human genomes
**DDBJ**	DDBJ (1987)	SRA (2009)	BioProject (2011)	BioSample (2013)	GEA (2018)	JGA (2013)
**EMBL-EBI**	European Nucleotide Archive (ENA) (originally EMBL Nucleotide Sequence Database, 1982)				Array Express (2001) / Expression Atlas	EGA (2008)
**NCBI**	GenBank (1982)	SRA (2008)	BioProject (2011)	BioSample (2011)	GEO (2001)	dbGaP (2007)

INSDC policy does not impose upon intellectual property rights. Publication of deposited sequences can be delayed until their journal publication, during which only journal editors and reviewers can access the information. Updates are accepted only from the original data providers so that appropriate recognition or credit can be traced. Thus, INSDC has been long recognized as the reliable framework for sustainably maintaining NSD and associated metadata throughout the scientific community. The framework became a model for the Open Access movement for academic literature (https://www.budapestopenaccessinitiative.org/), and was also the template of the FAIR principle for promoting Findable, Accessible, Interoperable, and Reusable data (https://www.force11.org/group/fairgroup/fairprinciples).

## INSDC POLICY

The INSDC supports deposition and distribution of data from publicly funded science, but does not claim the ownership of data. Data ownership is retained by the submitter. Although deposited data may be reformatted by the INSDC, original data providers retain rights to update records. Intellectual property rights are managed through patents and other measures independently from the INSDC although patented sequences are deposited within the INSDC by national or regional patent offices. The fundamental data-sharing policy of the INSDC was published in 2002 by our advisory board: free and unrestricted access to all of the data records, without use restrictions, licensing requirements, or fees on the distribution or use by any party (5). Another important principle is that data be permanently accessible as part of the scientific record including all updates. The same policy was adopted as the Bermuda Principles for the Human Genome Project in 1996, and as the Fort Lauderdale Agreement for genome sequencing data in 2003. The policy should not be misinterpreted as open access, where use restriction may apply depending on the licensing terms. The policy was reaffirmed by the advisory board in 2016 (6,7).

As the science that sequencing can support, INSDC databases have responded with extended and broader services and deeper integration with databases beyond INSDC. For example, in addition to traditional repositories (Table 1), the INSDC host data for gene expression including transcriptomes and microarrays. Since recent quantitative data rely on next-generation sequencers, expression information with metadata is registered in the functional genomics databases, Genomic Expression Archive (GEA) (2), Expression Atlas (8) and Gene Expression Omnibus (GEO) (9), while their raw sequence data are deposited in INSDC. To comply with informed consent agreements from donors, human genomes may be subject to access restrictions and are captured in sister databases operated by each partner that do not form part of INSDC: JGA at DDBJ (2), EGA at EMBL-EBI (10) and dbGaP at NCBI (11). Nonetheless, where appropriate, these databases share technical infrastructure and data standards with those of INSDC allowing interoperability.

## DATASIZE AND STORAGE

**Figure 1.**
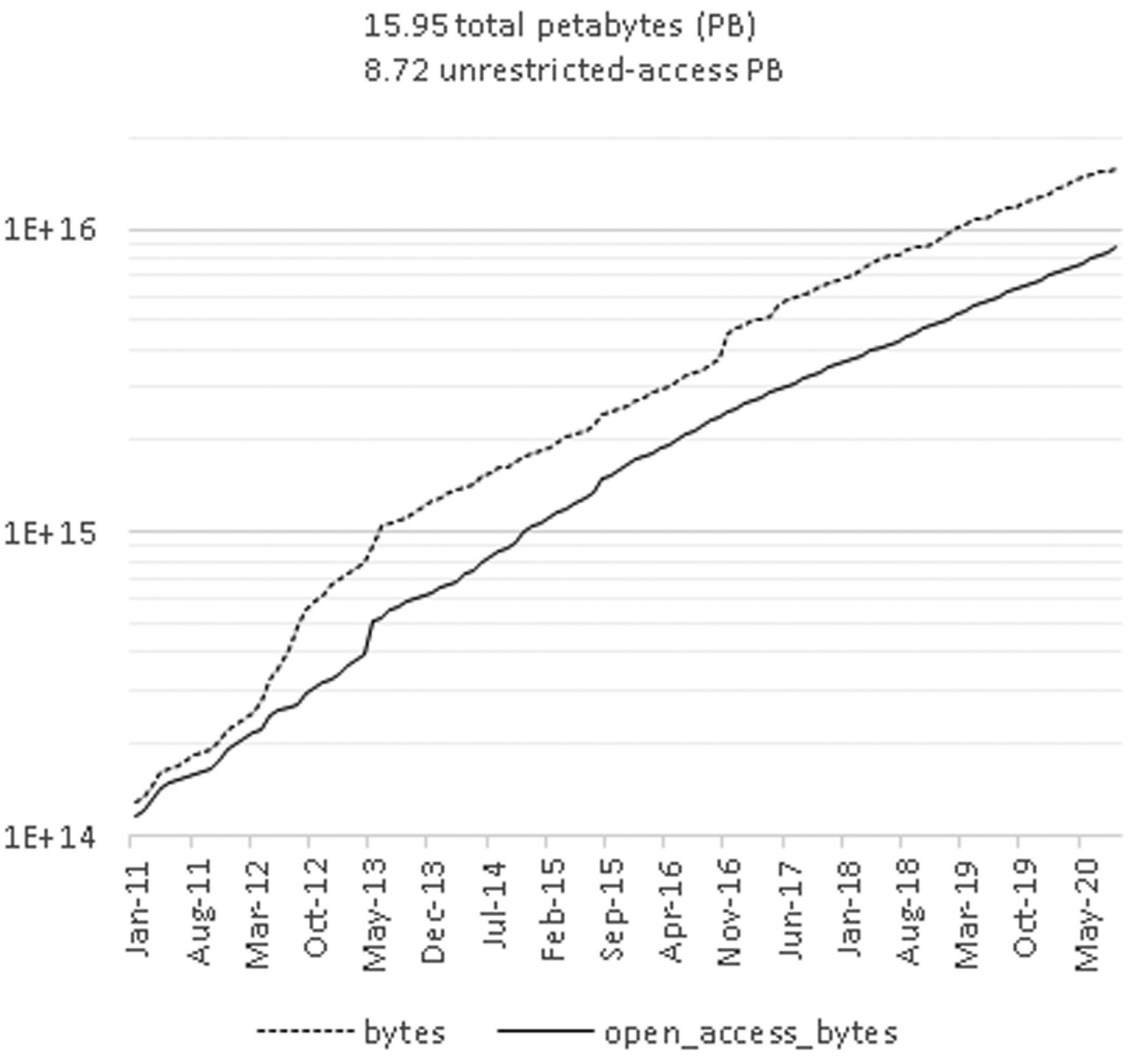
Cumulative 10-year growth of raw next generation sequence data: total bytes (dashed) and unrestricted-access bytes (solid).

The total size of sequence data maintained by the INSDC has exceeded 9 petabytes in 2020. Data grew roughly 10 times in the last 4-year period and the same growth is expected in the coming years. The increase is dominated by the raw next generation sequence data (Figure 1). The data size at NCBI surpasses 16 petabytes when access-restricted personal genomes are included. This exponential growth starts to affect usability by researchers in terms of data search, transfer and analysis; the size reduction is an imminent task. The current dominant raw data format includes base-quality score information. This information, however, is not always exploited in bioinformatic analyses. To reduce data size, NCBI is starting to offer read data without base-quality scores and APIs within the CRAM Data Compression framework are offered from EMBL-EBI (https://www.ga4gh.org/cram/). In addition, NCBI has shifted SRA data (https://www.ncbi.nlm.nih.gov/sra) to commercial cloud environments to improve data access and reduce computation cost for users. Commercial cloud environments offer storage options for different use frequencies: ‘hot’ storage for highly accessed data and less expensive ‘cold’ storage for older, less accessed studies. Meanwhile EMBL-EBI and DDBJ are more devoted to local, on-premises solutions. Since the two nodes mirror the complete NCBI data, users benefit from multiple choices depending on their computing environment.

## COLLABORATION

The three INSDC partners keep annual meetings to maintain data standards, formats and annotation quality. All results are summarized in the INSDC website (http://insdc.org/documents/); data providers are encouraged to check these documents for feature definitions and controlled vocabularies used in the sequence annotations. Deposition of sequence data in the integrated public archive is becoming ever more important as science covers a wide breadth of biological species, and as the scientific community interacts with the general society more deeply. Although the INSDC is supported by the host governments of its three members, its governance is independent from requests or needs of any specific political or scientific bodies, helping to ensure the collaboration's support of FAIR principles. This principle has been empirically proven as the reliable and sustainable way of data-sharing for the past 30 years. Sharing the full data between nodes is also important in cases of catastrophic failures or disasters.

## DATA TRACEABILITY

Nucleotide sequences are often the key information in life science studies. The INSDC offers metadata information to indicate the origin of bioresource using the BioSample repository and the /COUNTRY qualifier in the NSD annotation defined as ‘locality of isolation of the sequenced organism indicated in terms of political names for nations, oceans or seas, followed by regions and localities’. With these metadata with ANs, scientific origin and responsible authors can be fully clarified and used to discern the provenance of biological samples. Their geographical origin becomes important in terms of bioresource access and benefit sharing (ABS; https://www.cbd.int/abs/). The same is true for patented nucleotide sequences. At least in the United States, Europe, Japan and Korea, patent offices deposit sequences associated with patents which are published within the INSDC framework. Although INSDC partners do not track usage of individual sequences, the current system offers a realistic and viable solution for data traceability.

## COVID-19 RESPONSE

The global COVID-19 crisis is an eminent driver for rapid sharing of data. Without the FAIR data sharing scheme, rapid and timely analysis may be undermined. For this reason, publicly funded COVID-19 sequences from the United States and the European Union are registered in INSDC as well as in other repositories (e.g. GISAID at https://gisaid.org). The INSDC provides access to the genomic sequence data from SARS-CoV-2, the virus responsible for COVID-19 without restriction on use or distribution and without the need to register for access. The advantage of the INSDC is its support of raw sequence data to guarantee reproducibility, ability to support analysis of cross-species information, and deep interrogation with the public bioinformatics data infrastructure as a whole. In August 2020, the INSDC issued a public statement to ask all research communities to deposit raw and assembled sequence data to INSDC (http://www.insdc.org/documents). The core institutions have also established COVID-19-specific portals for access to COVID-19 data. The NCBI SARS-CoV-2 resource page (https://www.ncbi.nlm.nih.gov/sars-cov-2/) supports sequence data downloads, links to COVID-19 literature and registered clinical trials and genetic tests. The European COVID-19 Data Platform (https://www.covid19dataportal.org/) provides a data portal for all categories of information.

## FUTURE OUTLOOK

The data growth in population genomics and metagenomics is changing the landscape of sequence studies. Users can no longer conduct large-scale studies without cloud- or supercomputing. New sequences are coming from economically growing countries and data re-use and re-analysis are becoming common. Under this situation, a globally centralized and standardized framework is crucial because new discoveries arise from wider and statistical sequence comparisons and analyses. To this end, the INSDC supports the Genomic Standards Consortium (12) to build Minimum Information about any Sequence (MIxS, where x denotes ‘any’; https://gensc.org/mixs/) checklists for the collection of rich contextual metadata about biological samples and experimental technologies for a wide variety of genomic data. We encourage submitters to use these checklists when preparing the metadata for submission. We also recommend using the international protein nomenclature guidelines (https://www.ncbi.nlm.nih.gov/genome/doc/internatprot_nomenguide/) so that annotation ontologies are streamlined. As for personal information, our websites and submission processes are compliant with relevant data protection regulations. We also encourage submission of different types of analysis information such as methylation and genome maps. The INSDC is committed to integration and standardization of genome sequence data and their metadata for the benefit of not only science but all types of community worldwide.
